# Necrology

**Published:** 1899-01

**Authors:** 


### NECROLOGY.

At Norristown, Pa., January 2, 1899, Dr. Harry B. Rayner, aged sixty-nine years. Dr. Rayner was a self-made man in the broadest acceptance of the word. His place in the field of veterinary science was marked out through the apprenticeship system, having from childhood been trained in the atmosphere of veterinary practice by his father, a well-known English veterinarian, who early located in the immediate vicinity of Philadelphia. Dr. Rayner was the youngest of five brothers, who, up to the time of his death, were all in active practice, each of whom had nearly attained or passed the three-score-and-ten milestone in life, each of whom has won an enviable reputation in their respective communities and at large throughout our country among the profession wherever known. Dr. Harry B. Rayner was of a strong religious turn of mind, and was active in religious work in the community where he lived; never active in association work, but frequently attended association meetings, which he much enjoyed. Naturally of a retiring disposition, he found his greatest service for his profession in the field of his daily work, by steadfast devotion to his clients' interests, and in the relief of suffering among the lower animals.

AT Syracuse, N. Y., in December, Charles F. Ebner, V.S., graduate of the Ontario Veterinary College, class of 1894. Resolutions honoring the memory of Dr. Ebner and extending sympathy to his bereaved family were adopted by the veterinarians of Syracuse.

---

Dr. Nelson P. Hinkley, of Buffalo, N. Y., made a flying visit to Philadelphia in November. Dr. Hinkley recently disposed of his private practice to Messrs. Baker and Ryder, formerly of Cortland and Deposit, N. Y., respectively. Dr. Willyoung, formerly of the firm of Hinkley and Willyoung, will continue in private practice in that city.

